# Income, depressive symptoms, and anxiety symptoms in Indonesia: a two-year cross-lagged panel analysis of socioeconomic gradients

**DOI:** 10.3389/fpubh.2026.1839549

**Published:** 2026-07-31

**Authors:** Aryana Satrya, Mashita Fajri, Herni Susanti, Khusnul Aini, Helen Brooks, Penny Bee, Asri Maharani

**Affiliations:** 1 Centre for Social Security Studies, School of Strategic and Global Studies, Universitas Indonesia, Depok, Indonesia; 2 Department of Management, The Faculty of Economics and Business, Universitas Indonesia, Depok, Indonesia; 3 Mental Health and Psychiatric Nursing Research Group, Department of Psychiatric and Mental Health Nursing, The Faculty of Nursing, Universitas Indonesia, Depok, Indonesia; 4 Mental Health Research Group, Division of Nursing, Midwifery and Social Work, School of Health Sciences, University of Manchester and Manchester Academic Health Science Center (MAHSC), Manchester, United Kingdom

**Keywords:** anxiety symptoms, cross-lagged panel analysis, depressive symptoms, household income per capita, Indonesia

## Abstract

**Background:**

Socioeconomic status influences mental health, but few long-term studies explore how household income and psychological outcomes interact in low- and middle-income countries. This study examines the 2-year bidirectional relationship between household income per capita and depressive and anxiety symptoms in Indonesia.

**Method:**

Data from the STAND survey were analyzed using a two-wave cross-lagged panel design within an SEM framework. Two models were estimated: one for depressive symptoms (CES-D 10) and one for anxiety (GAD-7). Household income per capita (IDR) was categorized into lower- and higher-income group and used as the main socioeconomic indicator. Models adjusted for comorbidities, age, sex, education, employment status, and marital status. The sample included 18,164 respondents with complete data in 2023 and 2025.

**Results:**

At baseline, 4.4% reported depressive symptoms, and 8.5% reported anxiety. In the first model, baseline household income status predicted follow-up household income status (β = 0.361, *p* < 0.001) and fewer depressive symptoms (β = −0.014, *p* = 0.043). Those with baseline depressive symptoms were moderately likely to continue experiencing symptoms (β = 0.270, *p* < 0.001), which also predicted lower income later (β = −0.013, *p* = 0.039). In the second model, baseline household income status predicted follow-up household income status (β = 0.360, *p* < 0.001) and fewer anxiety symptoms (β = −0.035, *p* < 0.001). Baseline anxiety symptoms persisted (β = 0.257, *p* < 0.001), but did not significantly predict future income.

**Conclusions:**

Findings reveal a two-way link between household income status and depressive symptoms: lower household income status is associated with depressive symptoms, which then lower subsequent household income status. For anxiety, the connection is one-way: lower household income status is associated with worse symptoms, but not vice versa. These results highlight complex links between economic and mental health in Indonesia, emphasizing policies that address both poverty and mental health to break cycles.

## Highlights

- While socioeconomic gradients are clearly associated with mental health, there is limited evidence from LMICs, particularly from longitudinal studies.
- We employed STAND-Indonesia longitudinal data (*n* = 18,164; 94.4% retention) and used cross-lagged panel analysis to determine the directional relationships between household income status and mental health outcomes.
- Our analysis found that lower household income status predicts future depressive and anxiety symptoms, while depressive symptoms also predict lower subsequent household income status. Anxiety symptoms persist but do not predict income change.
- Poverty alleviation interventions in Indonesia, such as conditional cash transfers and national health insurance, could simultaneously serve as mental health interventions if made accessible to the most economically marginalized.
- Findings are restricted to Java, Indonesia's most developed island, so results may not generalize to other regions with different income levels, healthcare access, or cultural contexts.

## 1 Introduction

Depression and anxiety are among the leading causes of disability globally, generating substantial economic and social costs. The Global Burden of Disease Study 2023 reported marked increases in the age-standardized disability burden of both conditions since 2010, with anxiety disorders rising by 62.8% and depressive disorders by 26.3%, placing them among the top causes of years lived with disability worldwide (1). This burden is disproportionately concentrated in LMICs, where over 80% of people with common mental disorders reside and where these conditions account for nearly 9% of total disease burden, yet health systems remain severely under-resourced to respond (1, 2). Indonesia, the world's fourth most populous nation, exemplifies this challenge. An estimated 8.3 million Indonesians live with depression, and 12.4 million with anxiety, collectively contributing over 2.7 million DALYs (3), yet the treatment gap remains wide, driven by limited mental health infrastructure, low-income levels, and a rapidly aging population (4–6).

The negative association between socioeconomic status and mental health is well established, with lower income, limited education, and material deprivation consistently linked to higher rates of depression and anxiety (7–9). However, understanding the direction of this relationship remains a fundamental challenge. Two competing hypotheses have been proposed to explain the association between SES and mental health. First, the social causation hypothesis posits that the conditions of poverty, including stress, increased negative life events, worse physical health, reduced access to healthcare, and stigma, precipitate or maintain mental ill-health (10, 11). Conversely, the social drift (or social selection) hypothesis proposes that individuals living with mental illness drift into, or remain in, conditions of poverty as a result of increased health expenditure, reduced income, and lost employment (12).

Prior studies increasingly recognize that these two pathways are not mutually exclusive but operate simultaneously, creating a bidirectional and cyclical relationship between poverty and mental health (13, 14). Jiménez-Solomon and colleagues found that low SES and poor mental health create a cycle of cumulative disadvantage, leading to increasing mental health deterioration and socioeconomic decline over time (14). This bidirectional perspective is well-documented across multiple dimensions of SES: financial stress, food insecurity, and unstable housing significantly increase the risk of developing depression (social causation), while depression often leads to impaired occupational functioning, job loss, and financial decline, which can perpetuate poverty (15).

Despite growing recognition of this bidirectionality, most existing evidence comes from high-income countries, and the mechanisms and temporal dynamics of the income–mental health relationship in LMIC settings remain insufficiently understood (10, 12). Cross-sectional studies, which predominate in the LMIC literature (7, 16), are unable to disentangle the direction of these associations or address whether low income precedes poor mental health, whether mental ill-health depresses income, or both. For example, cross-national evidence from the WHO Study on Global Aging and Adult Health (WHO-SAGE), which studied nationally representative samples from six large LMICs (China, Ghana, India, Mexico, Russia, and South Africa), found that lower education, poverty, indebtedness, and past informal-sector occupation had significant positive associations with depression among older people, while pension support and health insurance showed significant negative associations (17). Furthermore, only a few studies include anxiety as an outcome alongside depression, and few apply analytical methods capable of capturing reciprocal effects over time.

In Indonesia specifically, longitudinal evidence on this relationship remains scarce. Most studies have relied on cross-sectional designs (7, 18, 19) or focused on specific subpopulations, such as adolescents (20), older adults (21), or women in urban areas (22), limiting inferences about temporal dynamics in the general adult population. One notable exception used longitudinal data from the Indonesia Family Life Survey and found that poor mental health was associated with reduced employment probability and lower community participation, even after accounting for potential endogeneity (11), lending empirical support to the social selection pathway in the Indonesian context. However, the reverse pathway, namely whether income influences subsequent mental health, and the simultaneous operation of both pathways, remain largely untested in Indonesia. Existing studies in Indonesia have largely examined only one side of the socioeconomic–mental health relationship. Previous research has shown that poverty, income shocks, and household consumption are associated with depression, suicide risk, and common mental disorders (22–24), while other studies have examined how poverty-related stress and anxiety may contribute to reduced productivity and economic decline (25).

Understanding these dynamics is particularly important in the Indonesian context, where rapid economic development coexists with persistent income inequality, and where mental health services remain underfunded and inaccessible to large segments of the population. Addressing these gaps, this study aims to examine the bidirectional associations between household income per capita and both depressive and anxiety symptoms among adults in Java, Indonesia. It will use cross-lagged panel analysis (CPLA) within a structural equation modeling framework on two waves of the STAND longitudinal household survey collected in 2023 and 2025. This approach allows us to model both the social causation and social selection pathways simultaneously, while accounting for the temporal stability of each variable, providing more rigorous longitudinal evidence than has previously been available in this setting.

## 2 Objective

This study aims to examine the bidirectional associations between household income per capita and depressive and anxiety symptoms among adults in Java, Indonesia, using cross-lagged panel analysis within a structural equation modeling framework.

## 3 Method

### 3.1 Participants and procedure

Data were obtained from the first wave in 2023 (i.e., baseline) and the second wave in 2025 (i.e., follow-up) of the Sustainable Treatment for Anxiety and Depression in Indonesia (STAND-Indonesia) study (26). It was conducted in four provinces on Java Island: Banten Province, West Java Province, Central Java Province, and East Java Province. The study design and sampling procedures for Wave 1 have been described in detail elsewhere (27), and identical procedures were applied at Wave 2 to ensure comparability across waves. At baseline, 19,236 individuals were surveyed. Of these, 18,164 were successfully re-contacted and re-surveyed in 2025, yielding a follow-up rate of 94.4%. Non-response occurred among 521 participants (5.42%), due to migration and loss to follow-up (205; 2.13%), death (236; 2.46%), and refusal to be interviewed (80; 0.83%). Longitudinal analyses were restricted to respondents who completed assessments at both waves (*n* = 18,164), while participants with baseline-only data were excluded from the cross-lagged panel analyses to ensure consistency of the analytic sample across time points.

Data collection for Wave 1 was conducted from 27 July to 20 October 2023, and Wave 2 from 1 September to 15 October 2025. Each survey wave lasted approximately 7–12 weeks, with interviews averaging around 30-45 min per participant.

### 3.2 Measures

#### 3.2.1 Household income per capita

At baseline (2023), household income was assessed by asking respondents to report their average monthly household income. A substantial proportion of responses (*n* ≈ 8,451) were missing due to “don't know” answers or skipped items in the computer-assisted personal interviewing (CAPI) system. Missing values in Wave 1 income were addressed using subgroup-specific single imputation based on employment status, education level, and urban/rural residence. This cell-based approach replaces missing values with the mean income observed among respondents within substantively defined socioeconomic subgroups and is commonly used in large-scale household surveys to improve predictive accuracy relative to unconditional mean substitution (28). Although single imputation is generally not recommended as a best-practice approach due to its tendency to attenuate sampling variability and underestimate standard errors (29, 30), it can still be applied operationally in certain contexts. In our study, imputations were informed by national survey experts with extensive field experience, ensuring that the filled-in values reflected contextual realities rather than purely statistical assumptions.

Following imputation, total household income was equivalized by dividing it by the number of household members to obtain household income per capita. Household income per capita was subsequently categorized into two groups based on the sample distribution to distinguish lower- and higher-income households for analysis.

At follow-up (2025), household income was assessed by summing reported monthly income from multiple sources (e.g., wages, business income, transfers). Missing data at follow-up were comparatively lower and were handled using the same single imputation procedure described for baseline. Household income per capita at follow-up was subsequently categorized into two groups based on the sample distribution for analysis. Households below the median income were classified as lower income, whereas those at or above the median were classified as higher income.

#### 3.2.2 Depressive symptoms

Depressive symptoms were assessed using the 10-item Center for Epidemiologic Studies Short Depression Scale (CES-D-10). This instrument has been employed in previous large-scale surveys in Indonesia (31). Eight items assessed negative symptoms, while two (items 5 and 8) measured positive symptoms. Participants rated how often they experienced each symptom “during the past week” on a four-point scale. The CES-D-10 scores range from 0 to 30, with the total score obtained by summing all item scores after reversing the positive mood items. This scale has demonstrated robust psychometric properties, such as reliable predictive accuracy and strong correlations with the original 20-item version, especially in community populations (32). The Indonesian version of CESD-10 demonstrates robust test-retest reliability and internal consistency, with an interclass correlation of 0.85 and a Cronbach's α of 0.86 in individuals aged 15 and above (33), and 0.9 among adolescents (34). In the STAND survey, the internal consistency of the CES-D 10 was lower than previously reported validation studies, with Cronbach's α of 0.566 at baseline (27) and 0.633 at follow-up. Confirmatory Factor Analysis (CFA) of the CES-D 10 has been reported elsewhere (27).

#### 3.2.3 Anxiety symptoms

The 7-item Generalized Anxiety Disorder (GAD-7) scale measures anxiety symptoms and is renowned for its high internal consistency and convergent validity, reflecting its reliability and accuracy. It evaluates symptoms such as nervousness, excessive worrying, difficulty relaxing, and restlessness, which are typical of GAD (35). The GAD-7 scale assesses anxiety symptoms on a range from 0 to 21. A previous study validated the GAD-7 among Indonesians, demonstrating strong internal consistency and reliability, with a Cronbach's α of 0.867 and validity coefficients ranging from 0.648 to 0.800 (*p* < 0.01) (36). The internal consistency of the GAD-7 in the STAND survey was acceptable (Cronbach's α = 0.770). CFA of the scale has been reported elsewhere (27).

### 3.3 Covariates

The covariates included comorbid (37), age (38), gender (39), education (7), employment status (40), and marital status (41). Age plays a significant role in mental disorders (38, 42) and was categorized into 18–24 years (reference), 25–34 years, 35–44 years, 45–54 years, 55–64 years, and ≤ 65 years. Comorbidities were summarized using the mean and standard deviation (SD). Sex is dichotomised into male and female. Education was divided into four categories: no education, primary school, junior high school, and senior high school or higher. Employment was categorized as employed and unemployed. Marital status was categorized into single, married, and widowed (which includes both divorced and separated).

### 3.4 Statistical analysis

We conducted univariate descriptive analyses to summarize participant characteristics at each survey wave. Categorical variables were presented as frequencies and percentages, while continuous variables were summarized using means and standard deviations (SD). Baseline descriptive statistics (Wave 1) were calculated using all available respondents surveyed in 2023, whereas follow-up descriptive statistics (Wave 2) were calculated using respondents retained at follow-up. To improve transparency, descriptive comparisons between waves were presented using Pearson's chi-square tests for categorical variables and independent-samples *t*-tests for continuous variables. These comparisons were intended to describe differences in sample composition across waves rather than serve as formal within-person longitudinal hypothesis tests.

The empirical analysis was conducted within a Structural Equation Modeling (SEM) framework, which allows for the simultaneous estimation of multiple interrelated pathways, accounts for measurement error, and provides model fit indices to assess adequacy. SEM is particularly suited for longitudinal epidemiological studies because it can incorporate latent constructs and test directional or reciprocal relationships across time (43).

Within this framework, we employed a cross-lagged panel analysis (CLPA) design (43). CLPA is a statistical method used to clarify bidirectional or directional relationships among variables over different time points. Cross-lagged panel models (CLPMs), also known as cross-lagged path models or cross-lagged regression models, utilize panel data that includes longitudinal observations, capturing data from individuals or points at multiple times (44). Specifically, we examined two CLPMs: the first model examines the relationship between household income per capita and depressive symptoms, while the second model analyses its association with anxiety symptoms. This approach allowed us to illuminate temporal relationships and potential predictive patterns between economic conditions and mental health outcomes.

We provided model fit statistics, including RMSEA, CFI, and TLI, with overall goodness-of-fit statistics reported in Table 1 below. Statistical analyses were conducted using Stata version 19 BE.

**Table 1:** Overall goodness-of-fit statistics of each model.

Fit index	Model	
	1A	1B
SRMR	0.004	0.004
RMSEA	0.018	0.021
CFI	0.989	0.985
NNFI (TLI)	0.969	0.960

### 3.5 Ethics statement

The STAND-Indonesia study was approved by the Ethics Committee of University of Manchester (2023-16515-30356), the University of Indonesia (KET-237/UN2.F12.D1.2.1/PPM.00.02/2022) and the Ministry of Internal Affairs of the Republic of Indonesia (400.5/5621/Polpum). Participants provided informed consent prior to enrolment, which outlined the study's purpose, objectives, and the expected role of participants to ensure transparency. They were assured that their responses would remain confidential and anonymous. Access to survey data was restricted to the research team and stored securely on a password-protected device.

### 3.6 Patients and public involvement statement

Patients and the public were actively involved in developing the questionnaire and the pilot testing phase of this study. During the questionnaire design process, we sought input from individuals representing the target population to ensure that the survey items were culturally appropriate, clearly understood, and relevant to the participants with lived experiences. A pilot study was conducted before the main data collection to assess feasibility, refine wording, and improve clarity, informed by feedback from community members and stakeholders. Their insights helped enhance the comprehensibility and applicability of the measures used in this study. Additionally, this study is reported in accordance with the STROBE guidelines, and the completed STROBE checklist is provided in Supplementary Table 1.

## 4 Results

Table 2 presents descriptive statistics for baseline (2023) and 2-year follow-up (2025). These comparisons were intended to describe differences in sample composition across waves rather than serve as formal within-person longitudinal hypothesis tests. At baseline, data were available for 19,236 respondents. Mental health outcomes were reported by 19,208 participants for depressive symptoms (*n* = 849; 4.4%) and by 19,219 participants for anxiety symptoms (*n* = 1,632; 8.49%). Participants reported a mean of 0.19 comorbid conditions (SD = 0.48). Sociodemographic information was complete for sex (*n* = 19,236; 100%) and residency area (*n* = 19,236; 100%), while age groups were available for 19,206 (99.8%), marital status for 19,229 (99.9%), and education for 19,224 (99.9%). Employment status was reported by all respondents (*n* = 19,236; 100%). Household per-capita income was available for 19,159 (94.4%), with a slightly greater proportion classified as lower-income households (50.2%) than higher-income households (49.8%).

**Table 2:** Sample Characteristics at Wave 1 (2023) and Wave 2 (2025).

Characteristics	Wave 1 (2023)^*^	Wave 2 (2025)^#^	*p*-value
Mental health outcomes			
Depressive symptoms, *n* (%)			< 0.001
Yes	849 (4.42)	656 (3.61)	
No	18,359 (95.58)	17,508 (96.39)	
Anxiety symptoms, *n* (%)			< 0.001
Yes	1,632 (8.49)	1,308 (7.20)	
No	17,587 (91.51)	16,856 (92.80)	
Health conditions			
Comorbids, mean (SD)	0.19 (0.48)	0.24 (0.55)	< 0.001
Sociodemographic			
Age (years), *n* (%)			< 0.001
18–24	2,691 (14.01)	1,779 (9.79)	
25–34	3,705 (19.29)	3,580 (19.71)	
35–44	4,064 (21.15)	3,797 (20.90)	
45–54	3,957 (20.60)	3,815 (21.00)	
55–64	2,791 (14.53)	2,953 (16.26)	
≥65	2,003 (10.41)	2,240 (12.34)	
Sex, *n* (%)			0.265
Male	8,646 (44.95)	8,059 (44.37)	
Female	10,590 (55.05)	10,105 (55.63)	
Marital Status, *n* (%)			< 0.001
Single	2,767 (14.39)	2,222 (12.23)	
Married	14,240 (74.05)	13,682 (75.32)	
Widowed	2,222 (11.55)	2,260 (12.44)	
Socioeconomic indicators			
Education, *n* (%)			0.058
No education	2,499 (13.00)	2,811 (15.51)	
Primary school	6,070 (31.58)	5,198 (28.68)	
Junior high school	3,768 (19.60)	3,444 (19.00)	
Senior high school or higher	6,887 (35.82)	6,671 (36.81)	
Residency area, *n* (%)			0.649
Urban	9,625 (50.04)	9,031 (50.28)	
Rural	9,611 (49.96)	9,133 (49.72)	
Employment status, *n* (%)			< 0.001
Employed	10,790 (56.09)	10,699 (58.90)	
Unemployed	8,446 (43.91)	7,465 (41.1)	
Household income per capita, *n* (%)			< 0.001
Lower-income household	9,114 (50.19)	9,083 (50.01)	
Higher-income household	9,045 (49.81)	9,081 (49.99)	

^*^Values based on available data, missing values excluded.

^#^Report the descriptive statistics for those re-interviewed.

At the 2025 follow-up, the analytic sample comprised 18,164 respondents. The prevalence of depressive symptoms decreased significantly from 849 (4.4%) to 656 (3.6%) (*p* < 0.001), while anxiety symptoms declined from 1,632 (8.49%) to 1,308 (7.2%) (*p* < 0.001). The mean number of comorbid conditions increased from 0.19 (SD = 0.48) at baseline to 0.24 (SD = 0.55) at follow-up (*p* < 0.001). The age distribution shifted toward older groups (*p* < 0.001), with the share aged 18–24 declining from 14.0 to 9.8%, while those aged 55–64 and 65–74 increased to 16.3% and 9.1%, respectively. Sex distribution, educational attainment, and residency area remained largely stable across waves (all *p* > 0.05). Marital status was dominated by married respondents (75.3%), with widowed individuals accounting for 12.4%. Marital status (*p* < 0.001) and employment status (*p* < 0.001) differed modestly between waves, with employment increasing to 58.9% at follow-up. Household income categories showed little change over time, with approximately the same proportion of respondents classified as lower-income and higher-income households at follow-up *p* < 0.001. Longitudinal models were based on all available data from respondents with complete information across both waves (*n* = 18,164).

**Figure 1:**
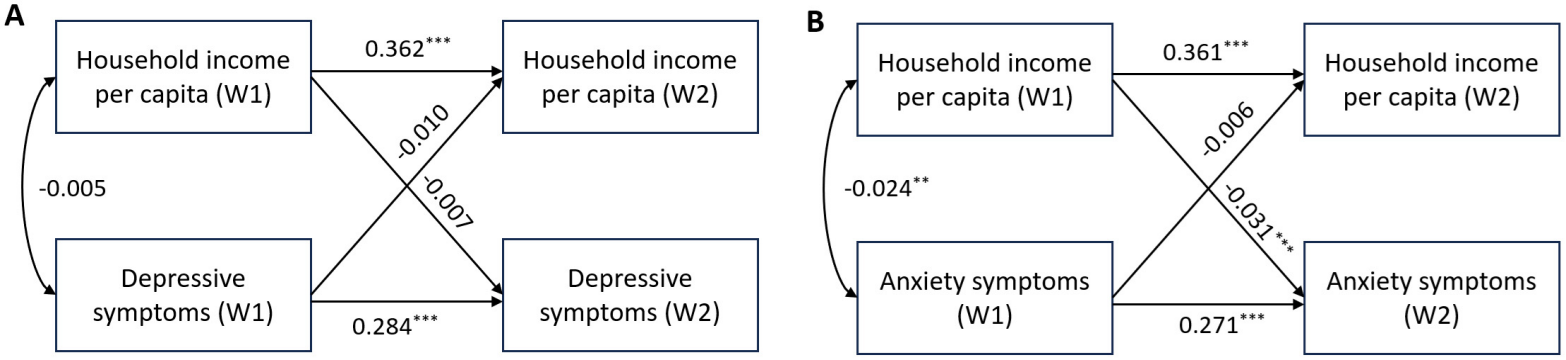
CLPA between household income per capita and mental health **(A)** depressive symptoms and **(B)** anxiety symptoms) across Wave 1 (2023) and Wave 2 (2025). W1, baseline (2023); W2, follow-up (2025), *p < 0.001, **p < 0.001, ***p < 0.001.

In this study, we conducted two cross-lagged panel analyses, each incorporating all covariates. In the first model (Figure 1A), after adjusting for comorbid, age, sex, education, employment status, and marital status, household income group at baseline was positively associated with household income group at follow-up (β = 0.361, *p* < 0.001) and negatively associated with depressive symptoms (β = −0.014, *p* =0.043). Baseline depressive symptoms significantly predicted depressive symptoms at follow-up (β = 0.270, *p* < 0.001), showing moderate temporal stability, and were negatively associated with household income per capita at follow-up (β = −0.013, *p* = 0.039).

In the second model (Figure 1B), the analysis showed that household income group at baseline was positively linked to household income group at follow-up (β = 0.360, *p* < 0.001) and negatively linked to anxiety symptoms (β = −0.035, *p* < 0.001), a similar pattern to model A. Baseline anxiety symptoms significantly predicted anxiety symptoms at follow-up (β = 0.257, *p* < 0.001), but were not significantly related to household income group at follow-up (β = −0.008, *p* = 0.205).

At baseline, household income group showed a significant negative covariance with depressive symptoms (β = −0.014, SE = 0.007, *p* = 0.049) and anxiety symptoms (β = −0.031, SE = 0.007, *p* < 0.001).

## 5 Discussion

This study examined the bidirectional associations between household income status and mental health outcomes over 2 years among adults in Java, Indonesia. The results support the social causation hypothesis, with lower baseline household income status predicting greater depressive and anxiety symptoms at follow-up, after adjusting for age, sex, marital status, employment status, and education. Both depressive and anxiety symptoms showed moderate temporal stability over the 2-year period, indicating that mental health difficulties tend to persist over time. The reverse pathway was also evident for depression, with poorer baseline mental health associated with lower household income status 2 years later, consistent with the social selection hypothesis. However, this reverse association was not observed for anxiety, where the association with subsequent household income status was not statistically significant. Sensitivity analyses with random-intercept specifications confirmed the robustness of the primary findings.

### 5.1 Comparison with existing literature: social causation vs. social selection

The finding that lower household income status predicted subsequent deterioration in both depression and anxiety aligns with a substantial body of evidence supporting the social causation pathway (10, 12, 45, 46). A study by Li and colleagues in China examined both pathways simultaneously using longitudinal data and found significant effects of poverty on depressive symptoms mediated through household living conditions, social participation, and life satisfaction, with depressive symptoms also directly predicting drift into poverty (46). Grounded in the stress-process model (47), this pathway posits that material deprivation elevates psychological distress through exposure to chronic stressors, including food insecurity, housing instability, and restricted access to healthcare (48). Our findings are consistent with the systematic review by Lund and colleagues, which demonstrated robust associations between poverty and common mental disorders across four LMICs, including Brazil, Chile, India, and Zimbabwe (10), and extend this evidence base to the Indonesian context, where comparable longitudinal data have been absent.

The evidence for social selection is more nuanced. The finding that depressive symptoms predicted lower household income status 2 years later is consistent with the well-established view that depression impairs cognitive functioning (49), reduces workplace productivity, and increases absenteeism (50, 51), thereby eroding earning capacity over time. This corroborates the only comparable Indonesian longitudinal evidence to date from Sibbald and colleagues, who found that psychological distress was associated with reduced employment probability and lower community participation, lending support to the selection pathway in this setting (11).

### 5.2 The asymmetry between depression and anxiety in predicting income

The absence of a significant reverse association for anxiety is noteworthy and has some precedent in the literature. Depression is typically associated with more severe and persistent functional impairment than anxiety (52), and its stronger links to reduced motivation, social withdrawal, and physical comorbidity may explain why it more consistently predicts income loss. Anxiety disorders, while disabling, may in some contexts be associated with maintained or heightened vigilance and continued work engagement, which could attenuate income effects in the short term (53).

An alternative explanation lies in the temporal dynamics of each condition. Anxiety disorders, particularly generalized anxiety, may exert their economic consequences more gradually and through more diffuse pathways, including increased healthcare utilization (54), interpersonal difficulties (55), and chronic fatigue, which may take longer to manifest as measurable income loss (56). The 2-year observation window employed in this study may therefore be better positioned to detect the more immediate and disruptive economic consequences of depression than the more insidious effects of anxiety. Longer follow-up periods across multiple waves will be important to determine whether the anxiety-to-income pathway eventually reaches significance as cumulative burden accumulates over time.

### 5.3 Implications for policy in the Indonesian context

The bidirectional findings carry important and distinct implications for Indonesian policy. The evidence for social causation, that lower household income status predicts subsequent deterioration in both depression and anxiety, suggests that poverty alleviation interventions can serve simultaneously as mental health interventions. Indonesia has expanded its social protection infrastructure considerably in recent years, including conditional cash transfer programmes (*Program Keluarga Harapan*) (57) and the near-universal coverage of the national health insurance scheme (*BPJS Kesehatan*), which by the end of 2024 covered more than 279 million people (58). These structural investments create an important platform. However, the mental health benefits of income-support programmes will only be realized if they are accessible to the most economically marginalized, and evidence suggests that the poorest Indonesians continue to face significant barriers to enrolment and benefit uptake (59).

The evidence for social selection, that depression predicts lower subsequent income status, adds a further, often overlooked layer to the policy argument. If depression erodes earning capacity through reduced productivity and absenteeism, then untreated depression functions not merely as a health problem but as a driver of deepening poverty. This creates a self-reinforcing cycle that income support alone cannot break. Indonesia's mental health system, however, remains severely under-resourced relative to the scale of need. Despite the formal inclusion of mental health services within BPJS Kesehatan's coverage framework, access remains constrained by a shortage of mental health professionals, geographic barriers, stigma, and widespread public unawareness that mental health expenses are covered at all (60). Under the current referral model, primary care facilities (*Pusat Kesehatan Masyarakat* or *Puskesmas*) serve as gatekeepers to mental health services, yet general practitioners working at this level frequently lack the training and confidence to identify and manage common mental disorders (61).

Our findings argue for a coordinated policy response that treats poverty alleviation and mental health interventions not as separate agendas but as mutually reinforcing. Integrating basic depression and anxiety screening into existing primary care platforms, particularly in lower-income communities, would allow early identification of those at greatest risk of the downward income-mental health spiral. Strengthening primary care workers' mental health literacy and reducing structural barriers to BPJS-covered mental health services would complement the economic protections already in place. Without such integration, social protection programmes risk being undermined by the unaddressed mental health burden that both drives and results from poverty.

### 5.4 Strengths and limitations

This study has several notable strengths. The use of a large, community-based longitudinal sample with a high follow-up retention rate of 94.4% reduces the risk of attrition bias and strengthens confidence in the temporal ordering of associations. The cross-lagged panel analysis within a structural equation modeling framework allows simultaneous estimation of both the social causation and social selection pathways within the same model and population, addressing a key methodological limitation of prior Indonesian studies, which examined only one direction. Inclusion of both depressive and anxiety symptoms as separate outcomes provides a more comprehensive picture of the income-mental health relationship than studies that focus on depression alone. The use of household income status derived from per capita household income as the exposure measure, rather than cruder proxies such as categorical income groupings or asset indices, provides an alternative indicator of socioeconomic position within the sample.

Several limitations should be acknowledged. First, the study is restricted to Java, the most densely populated and economically developed island in Indonesia, and findings may not generalize to other islands where income status, healthcare access, and cultural contexts differ substantially. Second, the two-wave design, while allowing directional inference, cannot fully establish causal relationships. CLPMs assume stationarity of effects across time and are sensitive to model specification; while random-intercept sensitivity analyses were conducted to address this, replication across three or more waves would provide stronger causal evidence. In addition, longitudinal analyses included only participants who completed both survey waves, and although follow-up retention (94.4%), attrition bias may exist if those lost differ systematically from retained participants. Third, household income at baseline had a high rate of missing data, which was handled through single imputation based on socioeconomic subgroup means. While this approach was informed by contextual expertise, single imputation does not preserve sampling variability and may underestimate standard errors, potentially affecting the precision of income-related estimates. Consequently, the magnitude of associations involving household income status should be interpreted with caution. Fourth, both mental health outcomes were assessed using self-report symptom scales rather than clinical diagnostic interviews, which may introduce measurement error and limit comparability with studies using diagnostic classifications. In addition, the CES-D0's internal consistency was lower than in validation studies, suggesting reduced reliability and weaker associations with depressive symptoms. Therefore, interpret findings cautiously. Finally, although covariates including comorbidities, age, sex, education, employment status, and marital status were included, residual confounding from unmeasured variables, such as life events and social support, cannot be ruled out.

## 6 Conclusion

This study provides evidence of bidirectional associations between household income status and common mental disorders in Java, Indonesia, with lower household income status predicting subsequent depressive and anxiety symptoms, and depressive symptoms in turn predicting lower subsequent household income status. The asymmetry between depression and anxiety in the reverse pathway points to clinically meaningful differences in how these conditions translate into economic disadvantage, and highlights the need for longer follow-up studies to capture the more diffuse economic consequences of anxiety disorders. For policy, these findings underscore that poverty alleviation and mental health interventions are not parallel agendas but mutually reinforcing ones: Indonesia's existing social protection infrastructure and primary care network offer a viable platform for integrated approaches, but realizing this potential will require sustained investment in mental health workforce capacity and the reduction of structural barriers to care.

## Data Availability

The data analyzed in this study is subject to the following licenses/restrictions. The data analyzed in this study are owned by the STAND-Indonesia project and are subject to project-specific access restrictions. Requests to access these datasets should be directed to Data are available upon reasonable request. Individual interested in using the data used in this study, may submit a protocol for a proposed paper to jemma.elston@manchester.ac.uk, who will inform the Authorship Group members (Professor PB, Professor HB, and Professor HS) of the proposed paper via email.
